# Building Interest in the Primary Care Specialty Through Enhanced Global Health Experience

**DOI:** 10.7759/cureus.50216

**Published:** 2023-12-09

**Authors:** Mark Hernandez, Michael O Ibiwoye, Melinda Ledbetter, Richard Thacker, Sebastian Diaz

**Affiliations:** 1 Medical Education, Quillen College of Medicine, Johnson City, USA; 2 Anatomy Department, Alabama College of Osteopathic Medicine, Dothan, USA; 3 Clinical Sciences, Alabama College of Osteopathic Medicine, Dothan, USA; 4 Medical Education, Northeast Ohio Medical University, Rootstown, USA

**Keywords:** higher education medical training, international and travel medicine, global health education, gender, primary care

## Abstract

Background

An overwhelming majority of matriculating medical students in the USA are keen to deliver quality health care to all people, including the socioeconomically disadvantaged populations in remote, resource-scarce regions nationally and worldwide. Here, we describe a protocol developed to evaluate the interest of our medical students in global health activities. We also examined the relationship between students' interest in global health and readiness to pursue a future career in the primary care specialty.

Materials and methods

We designed a survey in Qualtrics online software and reached all first-year and third-year medical students between 2019 and 2022 enrolled at the Alabama College of Osteopathic Medicine (ACOM). The survey utilized ordinal scale items to explore the medical students' interest in primary care residency programs, their interest in global health and international travel, and their perceptions of how a range of factors might motivate their desire to participate in global health activities. The study was approved by ACOM's Institutional Review Board (IRB). In order to compare findings from this study with data from other medical schools, we developed constructs using the national aggregate data, in percentages, from matriculants and graduates of Doctor of Osteopathic Medicine (DO) degree-granting medical schools according to gender, published by the American Association of Colleges of Osteopathic Medicine (AACOM). Statistical analysis of national aggregate data was performed using the unpaired t-test.

Results

Both female and male participants had lived or traveled abroad before starting medical school. Female (98%, n=249) and male (95%, n=140) participants in the first-year cohorts considered helping the underserved population as important or very important as it is related to a career in medicine. Females in the third-year cohorts (97%, n=71) also ranked this statement as important or very important compared to male cohorts (89%, n=31). A higher proportion of females (43%, n=108) compared to males (35%, n=52) in first-year cohorts agreed or strongly agreed that they would likely pursue a residency in primary care. More females (59%, n=43) than males (46%, n=16) in the third-year cohorts agreed or strongly agreed with the same statement. Analysis of the aggregate national data (2009-2022) revealed that the percentage (actual count not available) of female students who planned* *to practice in underserved/shortage area was higher both at the time of matriculation (M=51%, SD=4%) and before graduation (M=40%, SD=4%) compared to males (matriculation: M=40%, SD=5%; graduation: M=33%, SD=4%) presenting a significant difference (matriculation t(24)=6.7, p<0.0001; graduation t(24)=5.4, p<0.0001). Furthermore, a higher percentage of females at the time of matriculation (M=25%, SD=5%) and graduation (M=40%, SD=6%) planned* *to practice in the primary care specialties compared to males (matriculation: M=17%, SD=4%; graduation: M=29%, SD=6%) presenting a significant difference (matriculation: t(24)=4.6, p = 0.0001; graduation: t(24)=4.8, p<0.0001).

Conclusions

Interest in global health activities may be associated with interest in pursuing a future career in the primary care specialty. In this study, more female medical students expressed interest in participating in global health experiences, serving the underserved population domestically and abroad, and expressing interest in primary care than males.

## Introduction

The USA is facing a physician shortage in the rural and underserved areas of the nation [1]. By 2034, the nation is projected to face a shortage of primary care (PC) physicians of 17,800 to 48,000 [2]. Although, according to the National Residency Matching Program (NRMP), in 2022, there was a record number of first-year PC positions offered (36,227), 64.6% were filled by US seniors [3]. US Doctor of Osteopathic Medicine (DO) seniors saw a gain in the numbers who matched into family medicine, internal medicine, and pediatrics, still, the match in 2022 saw a "slight" decline in US seniors matching into PC compared to 2021 [3]. PC consists of family medicine, general internal medicine, general pediatrics, and geriatric medicine, and these physicians play a critical role in the health care of our nation and have an integral role in triaging and testing patients during the responses to the COVID-19 pandemic [4]. Understanding how medical students' interest in working in underserved areas is shaped during undergraduate medical education (UGM) can be helpful in developing tactical interventions throughout the medical school curriculum. Commitment to helping the underserved is usually higher when students begin medical education [5], and early tactical interventions can also help encourage more students to pursue residency programs in rural or underserved areas in PC specialties later. For example, participants in global health (GH) electives were more likely to pursue careers with underserved populations [6], and participation was also related to interest in PC [7]. Our previous observations on student participation in osteopathic medical outreach experiences abroad [8] suggested that students interested in GH would have other unique interests or past experiences that would help us better plan for international medicine (IM) elective rotations. To learn more about the student motivations, we designed a survey to gauge interest in a range of factors that might motivate their desire to participate in global health activities (e.g., being prepared to work in global health, functioning as a physician in underserved areas abroad, learning another language or learning more about cultural diversity). With the same survey, we also sought to identify if a correlation existed between gender and interest in serving the underserved and/or interest in PC. 

## Materials and methods

We developed a web-based survey (see Appendix) using Qualtrics (Qualtrics, Seattle, US) online software. The survey was approved by the Alabama College of Osteopathic Medicine (ACOM) Institutional Review Board (IRB# HS220701-EX Interest in Global Health). The survey was administered to first-year osteopathic medical students (OMS I) and third-year osteopathic medical students (OMS III) starting in July 2019. The survey was distributed via email during the first week of classes, was voluntary, and used ordinal scale items to explore the medical student interest in PC residencies, their interest in GH and international travel, and their perceptions of how various factors which we thought might encourage/discourage their intent to participate in GH programs. To help ensure anonymity, the only demographic information asked for was gender. Only one survey could be completed by participants. A separate reminder, via mass email, was sent to each cohort to complete the survey, which stayed open for a week. Surveys were not sent later in the semester as our previous experience has shown that survey participation decreases after the first week of classes.

Altogether, 1105 participants were asked to complete the online survey. Between 2019 (n=183), 2020 (n=205), 2021 (n=186), and 2022 (n=208), a total of 782 subjects in four separate OMS I classes received the invitation to participate in the study. A total of 401 respondents completed the survey in the OMS I classes, yielding an overall average of 52% response rate (in 2019, it was 55%, n=101; in 2020, it was 50%, n=103; in 2021, it was 62%, n=115; and in 2022 it was 39%, n=82). The same questionnaire was also distributed to the OMS III classes, which yielded a total response of 161. In 2019, the OMS III class, which began medical school in 2017, yielded a 12% (n=19) response rate. In 2020, the OMS III class, which began medical school in 2018, yielded a 15% (n=23) response rate. For 2021 (17%, n=29) and 2022 (19%, n=37), both cohorts had seen a similar survey prior as OMS I students. We had no mechanism to determine if those who completed the survey in 2019 as OMS I students also completed a later survey in 2021 as OMS III students as we did not collect personal identifier data. For purposes of data analysis and reporting, the OMS II cohorts were excluded since we could not account for how many of them already took the survey during the previous year.

We analyzed reports from the American Association of Colleges of Osteopathic Medicine (AACOM), which provided aggregate data for matriculants and graduates of DO-granting medical schools, to identify national trends in gender and interest in PC in osteopathic medical schools [9,10]. The reports include information about topics of relevance to undergraduate medical education (UME),** **except for information on global health or international travel. For this study, the aggregate data published between 2009-2021 was used to help develop single unified constructs for percentage of students who plan to practice in underserved/shortage areas(see Table 22 in most recent Entering Student Survey [9], and Table 51 in most recent Graduating Senior survey [10]) and percentage of students who plan to practice in primary care specialties (see Table 28 in most recent Entering Student Survey [9] and Table 57 in most recent Graduating Senior Survey [10]). Data in both reports are also available based on gender. Data were analyzed, and plots and constructs were developed using GraphPad Prism version 8.4.3(686) (GraphPad Software, La Jolla, US). Statistical analysis involved an unpaired t-test assuming the Gaussian distribution method using MS Excel and verifying with GraphPad software.

## Results

Surveys were administered separately starting in July 2019 during the first week of fall classes. A new survey was subsequently conducted around the same time in 2020, 2021 and 2022. We did not include statements specific to the pandemic, as the same survey was used throughout the four-year period. Our analysis of the data suggests that COVID-19 impacted perceptions in a complex manner, and additional years of data may be necessary to see the overall impact of the pandemic on participant perception. The first item in our questionnaire asked the participants the following: With which of the following do you primarily identify? The replies included female, male, neither, prefer not to answer. Over the span of four years (2019-2022), the participants included females (n=254), males (n=147), preferred not to answer (n=0), and neither (n=0). One participant left this item blank. 

Table 1 (for OMS I) shows the percentages (and number of responses) according to gender. First-year female respondents, compared to first-year male respondents, were more likely to select "very Important" or "important" (combined) for every item in the survey when asked, "For each of the following items, please rate the relative importance of each item as it relates to your career in medicine". When asked, "For each of the following items, please select the response that best reflects your level of agreement", female respondents, compared to males, were more likely to select "strongly agree". Except for items "An international experience during medical school will make me more competitive for residency application" and "My religious and/or spiritual community encourages its members to serve on international mission trips" where a higher percentage of males selected "strongly agree", compared to females. The cost of travel would discourage travel internationally the most for both genders.

**Table 1:** Osteopathic medical students (OMS I) participated in the survey according to gender

Survey respondents (n=401)	For each of the following items, please rate the relative importance of each item as it relates to your career in medicine	Being prepared to work in global health	Being able to function as a physician in international healthcare settings	Being able to function as a physician in underserved areas in other countries	Learning a language other than English	Developing communication skills to better serve foreign-born patients	Gaining clinical experiences in international settings	Broadening my worldview	Learning more about cultural diversity	Helping the underserved	Learning how medicine is practiced outside the USA	For each of the following items, please select the response that best reflects your level of agreement	I would like to participate in a global health experience sometime during medical school	I would like to engage in international travel while in medical school	I would like to travel internationally after I finish medical school	I would like to volunteer as a physician in international settings after I complete medical school	My religious and/or spiritual community encourages its members to serve on international mission trips	Medical students in the USA need to experience how medicine is practiced internationally	Medical students in the USA need to learn how to serve patients from other countries	International travel provides medical students career advantages	An international experience during medical school will make me more competitive for residency application	I will likely pursue a residency in Primary care (i.e. family medicine, general internal medicine, or pediatrics)	Medical students in the USA need to learn how to serve patients from other ethnic cultures.	For each of the following items, please rate the extent to which they would prevent you from traveling	Unanticipated risks / adventure	Living conditions different from USA	Foods different from those in USA	Cultural Norms different from those in USA	Risk of disease	Risk of injury	Cost of travel	Being in Situations where I have only myself to rely on
Female (n=254)	Very important or important	189	208	207	212	240	202	247	245	249	217	Strongly agree	233	223	232	208	88	199	233	202	158	108	248	This factor would encourage me to travel internationally	88	76	175	156	2	1	2	35
		74%	82%	81%	83%	94%	80%	97%	96%	98%	85%		92%	88%	91%	82%	35%	78%	92%	80%	62%	43%	98%		35%	30%	69%	61%	1%	0%	1%	14%
Male (n=147)		91	93	99	116	130	99	135	136	140	110		118	117	132	114	55	99	129	109	97	52	139		73	51	111	94	6	5	5	43
		62%	63%	67%	79%	88%	67%	92%	93%	95%	75%		80%	80%	90%	78%	37%	67%	88%	74%	66%	35%	95%		50%	35%	76%	64%	4%	3%	3%	29%
Female (n=254)	Somewhat important	54	41	37	33	12	42	4	6	3	35	Neutral	16	22	17	36	126	49	18	44	57	97	5	Neither	131	142	70	89	126	148	84	149
		21%	16%	15%	13%	5%	17%	2%	2%	1%	14%		6%	9%	7%	14%	50%	19%	7%	17%	22%	38%	2%		52%	56%	28%	35%	50%	58%	33%	59%
Male (n=147)		45	45	39	24	12	40	8	7	4	26		22	19	10	24	68	37	13	31	43	49	6		63	84	29	51	83	92	46	84
		31%	31%	27%	16%	8%	27%	5%	5%	3%	18%		15%	13%	7%	16%	46%	25%	9%	21%	29%	33%	4%		43%	57%	20%	35%	56%	63%	31%	57%
Female (n=254)	Not at all important	10	4	8	7	1	9	2	1	1	1	Strongly disagree	4	8	4	9	38	5	2	7	6	48	0	This factor would discourage me to travel internationally	34	35	8	8	125	103	167	69
		4%	2%	3%	3%	0%	4%	1%	0%	0%	0%		2%	3%	2%	4%	15%	2%	1%	3%	2%	19%	0%		13%	14%	3%	3%	49%	41%	66%	27%
Male (n=147)		9	6	6	4	2	5	1	1	0	8		6	10	4	8	23	10	4	6	6	45	1		11	12	6	2	58	49	96	20
		6%	4%	4%	3%	1%	3%	1%	1%	0%	5%		4%	7%	3%	5%	16%	7%	3%	4%	4%	31%	1%		7%	8%	4%	1%	39%	33%	65%	14%

Table 2 (for OMS III) shows the percentages (and number of responses) according to gender. Third-year female respondents, compared to third-year male respondents, were more likely to select "very important" or "important" (combined) for every item in the survey when asked, "For each of the following items, please rate the relative importance of each item as it relates to your career in medicine". When asked, "For each of the following items, please select the response that best reflects your level of agreement female respondents", females were more likely to select "strongly agree", compared to males. Except for items "An international experience during medical school will make me more competitive for residency application" and "My religious and/or spiritual community encourages its members to serve on international mission trips", where a higher percentage of males selected "strongly agree", compared to females. The cost of travel would discourage travel internationally the most for both genders.

**Table 2:** Osteopathic medical students (OMS III) participated in the survey according to gender

Survey respondents (n=108)	For each of the following items, please rate the relative importance of each item as it relates to your career in medicine	Being prepared to work in Global Health	Being able to function as a physician in international healthcare settings	Being able to function as a physician in underserved areas in other countries	Learning a language other than English	Developing communication skills to better serve foreign-born patients	Gaining clinical experiences in international settings	Broadening my worldview	Learning more about cultural diversity	Helping the underserved	Learning how medicine is practiced outside the USA	For each of the following items, please select the response that best reflects your level of agreement.	I would like to participate in a Global Health Experience sometime during medical school.	I would like to engage in international travel while in medical school	I would like to travel internationally after I finish medical school	I would like to volunteer as a physician in international settings after I complete medical school	My religious and/or spiritual community encourages its members to serve on international mission trips	Medical students in the USA need to experience how medicine is practiced internationally	Medical students in the USA need to learn how to serve patients from other countries	International travel provides medical students career advantages	An international experience during medical school will make me more competitive for residency application	I will likely pursue a residency in primary care (i.e. family medicine, general internal medicine, or pediatrics)	Medical students in the USA need to learn how to serve patients from other ethnic cultures	For each of the following items, please rate the extent to which they would prevent you from traveling	Unanticipated risks / adventure	Living conditions different from USA	Foods different from those in USA	Cultural norms different from those in USA	Risk of disease	Risk of injury	Cost of travel	Being in situations where I have only myself to rely On
Female (n=73)	Very important or important	62	62	62	62	69	65	72	71	71	63	Strongly agree	64	66	70	64	28	57	68	49	37	43	70	This factor would encourage me to travel internationally	25	25	51	46	0	1	2	16
		85%	85%	85%	85%	95%	89%	99%	97%	97%	86%		88%	90%	96%	88%	38%	78%	93%	67%	51%	59%	96%		34%	34%	70%	63%	0%	1%	3%	22%
Male (n=35)		18	24	23	25	31	23	29	25	31	23		29	29	33	27	10	20	29	20	16	16	31		18	15	27	20	1	1	1	11
		51%	69%	66%	71%	89%	66%	83%	71%	89%	66%		83%	83%	94%	77%	29%	57%	83%	57%	46%	46%	89%		51%	43%	77%	57%	3%	3%	3%	31%
Female (n=73)	Somewhat important	10	10	10	9	4	8	1	2	2	10	Neutral	8	5	2	7	38	14	3	23	22	15	2	Neither	41	45	21	26	44	41	28	41
		14%	14%	14%	12%	5%	11%	1%	3%	3%	14%		11%	7%	3%	10%	52%	19%	4%	32%	30%	21%	3%		56%	62%	29%	36%	60%	56%	38%	56%
Male (n=35)		16	10	10	9	4	10	6	7	4	9		6	6	2	7	20	9	6	13	18	10	4		15	18	8	15	22	22	7	19
		46%	29%	29%	26%	11%	29%	17%	20%	11%	26%		17%	17%	6%	20%	57%	26%	17%	37%	51%	29%	11%		43%	51%	23%	43%	63%	63%	20%	54%
Female (n=73)	Not at all important	1	1	1	1	0	0	0	0	0	0	Strongly disagree	1	2	1	2	7	2	2	1	3	15	1	This factor would discourage me to travel internationally	7	3	1	1	29	31	43	16
		1%	1%	1%	1%	0%	0%	0%	0%	0%	0%		1%	3%	1%	3%	10%	3%	3%	1%	4%	21%	1%		10%	4%	1%	1%	40%	42%	59%	22%
Male (n=35)		1	1	2	0	0	2	0	2	0	3		0	0	0	1	5	5	0	2	1	9	0		2	2	0	0	12	12	27	5
		3%	3%	6%	0%	0%	6%	0%	6%	0%	9%		0%	0%	0%	3%	14%	14%	0%	6%	3%	26%	0%		6%	6%	0%	0%	34%	34%	77%	14%

**Figure 1:**
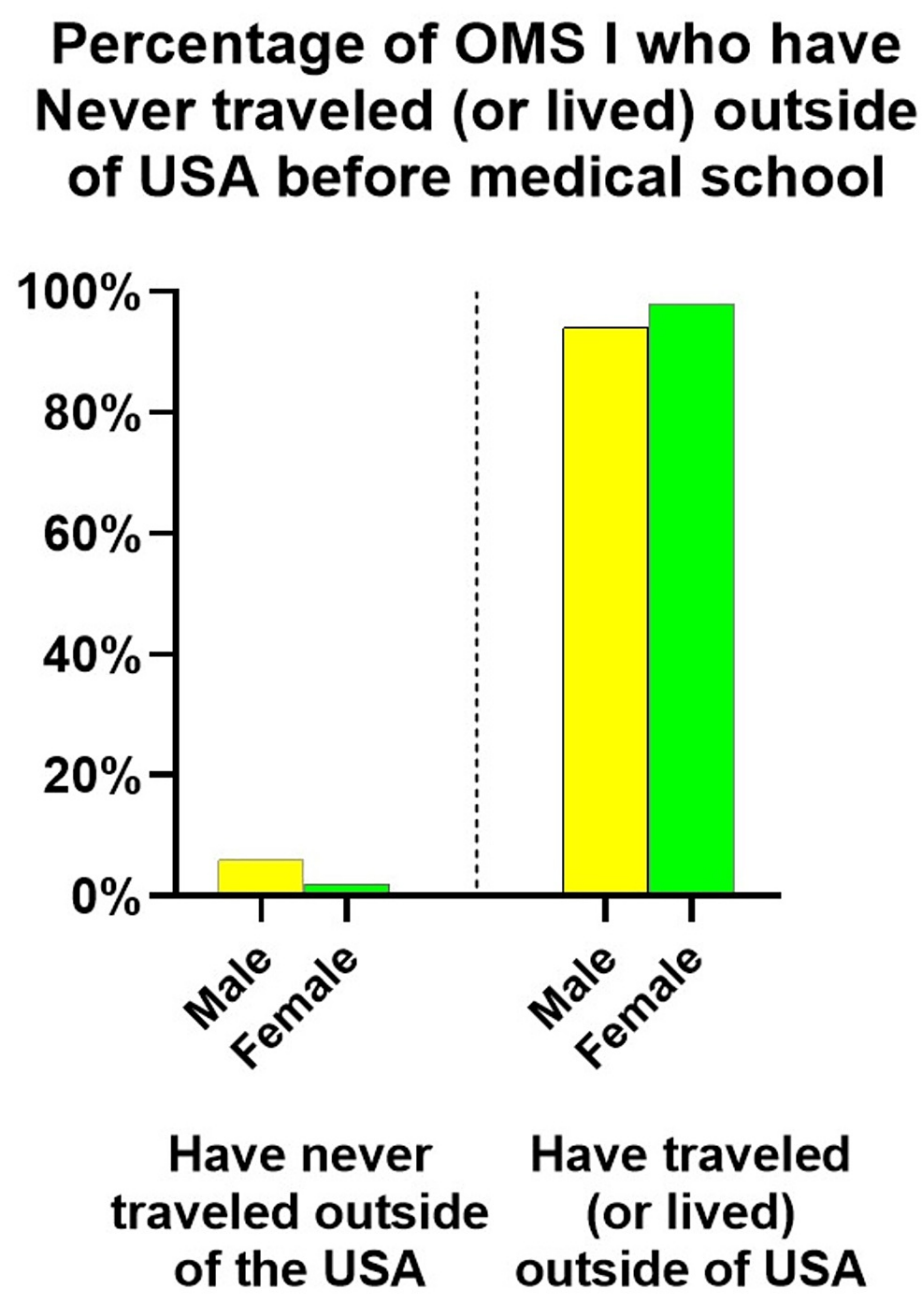
Most survey respondents had traveled abroad

Our survey asked to describe past experiences, such as traveling or living outside of the United States, and whether the participant spoke another language. Our survey results showed that 98% (n=249) of OMS I female and 94% (n=141) of male participants had lived or traveled abroad. Out of the total count, 2% (n=7) of female participants and 6% (n=9) of male participants had never left the country (see Figure 1). All other OMS I respondents selected more than one of the other choices: (a) I have traveled outside the USA for personal reasons, (b) I have traveled outside the USA as part of an educational experience, (c) I have traveled outside the USA as part of mission work related to my place of worship or (d) I have traveled outside the USA as part of a medical service trip (e) I have lived outside the USA, (f) I have studied another language other than English and/or (g) I have achieved fluency in a language other than English. 

Further, the results and discussion will focus on the specific items presented in Tables 1 and 2, where the responses could be compared to the national aggregate data. 

**Figure 2:**
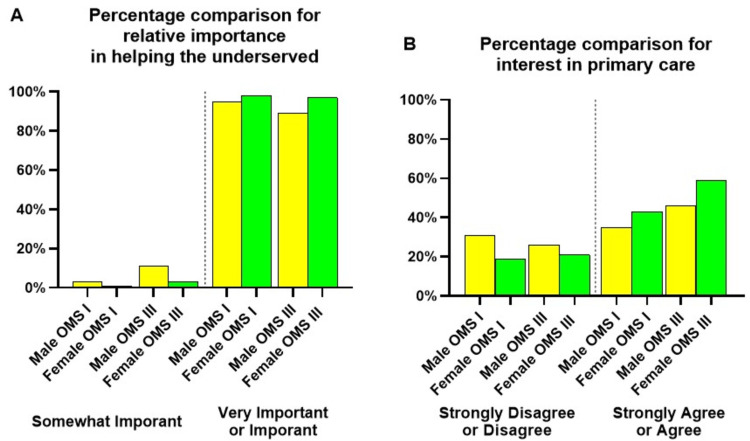
The relative importance of "helping the underserved" as it relates to a career in medicine (A) and "I will likely pursue a residency in primary care (i.e., family medicine, general internal medicine, or pediatrics)" (B)

Participants in global health electives were more likely to pursue careers with underserved populations [6]. When asked, "For each of the following items, please rate the relative importance of helping the underserved as it relates to your career in medicine", a higher proportion of female participants in OMS I and OMS III cohorts selected "very important" or "important" compared to male participants (Figure 2A). When asked, "I will likely pursue a residency in primary care (i.e., family medicine, general internal medicine, or pediatrics)", a higher proportion of female participants in OMS I and OMS III cohorts selected "strongly agree" or "agree" compared to male participants (Figure 2B).

Helping the underserved ranked as "very important" or "important" for OMS I female survey participants (98%, n=249) and male survey participants (95%, n=140). A higher proportion of OMS III females (97%, n=71) also selected "very important" or "important" compared to males (89%, n=31). Except for the OMS III 2019 cohort (Figure 2A), all other OMS III cohorts could have completed a similar survey when they were part of the OMS I or II classes during the previous years. In 2019, in the OMS III cohort, only 19 females and four males completed the survey. In 2020, the OMS III survey yielded the participation of 18 females and five males, who could have also participated in the 2019 survey as OMS II. OMS III 2021 (14 females, 15 males) and 2022 cohorts (26 females and 11 males) saw a small rise in participants. These changes are consistent with a class size increase starting in 2020 at the institution. 

Participation in global health experiences in low-income countries is associated with interest in primary care [7]. This observation led us to analyze the following item in our survey further: "I will likely pursue residency in primary care (i.e., family medicine, general internal medicine, or pediatrics)" (Figure 2B). A higher proportion of females (43%, n=108) compared to males (35%, n=52) in all OMS I cohorts strongly agreed or agreed with this statement. Similarly, a higher proportion of females in OMS III cohorts (59%, n=43), compared to males (46%, n=16), also strongly agreed or agreed with this statement.

**Figure 3:**
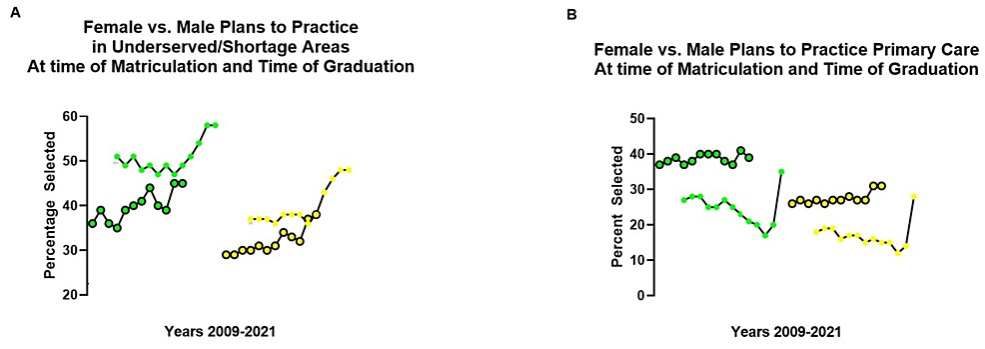
Constructs developed by plotting selected data found in the Academic Year Graduating Seniors Survey Reports, and Academic Year Entering Student Survey Reports published by AACOM Construct A: the percentage of entering class (solid dots) matched to the graduating class of seniors four years later (circles) who "Plan to practice in underserved/shortage areas"* a*ccording to gender (female green, male yellow). Construct B: the percentage of entering class (solid dots) matched to the graduating class of seniors (circles) who "Plan to practice in primary care specialties" according to gender (female green, male yellow). The constructs were developed by plotting selected data found in the Academic Year Graduating Seniors Survey Reports, and Academic Year Entering Student Survey Reports published by AACOM in the years between 2009 and 2021. Ten points were matched between entering and graduating cohorts four years later. Entering classes (2006-09) was not available; similarly, points for graduating classes after 2021 were not included, since data was not yet available when constructs were developed.

The AACOM does not collect information on global health-related activities or international travel [10]; therefore, it is difficult to compare how survey participants responded to the other items we asked in our survey. However, the item about planning to practice in underserved/shortage area is found in satisfaction instruments used at the time of matriculation for entering medical students and also before graduation [9,10]. In addition, this data is presented according to gender (Tables 3 and 4, Figure 3). 

**Table 3:** The percentage of students who plan to practice in underserved/shortage areas at the time of matriculation and before graduation according to gender (years 2009-2022) Selected data were found in the Entering Student Survey Reports (completed after matriculation) and the Academic Year Graduating Seniors Survey Reports (completed before graduation four years later). Data reports used here were published by AACOM between 2009 and 2022.

Item	Source	Gender	Mean	SD	Unpaired t-test	p-value
Plan to practice in underserved/shortage areas	Academic Year Entering Student Survey Summary Reports published between 2009 and 2022	Female	51%	4%	t(24)=6.6765	<0.0001
		Male	40%	5%		
	Academic Year Graduating Seniors Survey Summary Reports published between 2009 and 2022	Female	40%	4%	t(24)=5.3921	<0.0001
		Male	33%	4%		

**Table 4:** The percentage of students who plan to practice primary care at the time of matriculation and before graduation (years 2009-2022) Selected data were found in the Entering Student Survey Reports (completed after matriculation) and the Academic Year Graduating Seniors Survey Reports (completed before graduation four years later). Data reports used here were published by AACOM between 2009 and 2022.

Item	Source	Gender	Mean	SD	Unpaired t-test	p-value
Plan to practice in primary care specialties	Academic Year Entering Student Survey Summary Reports published between 2009 and 2021	Female	25%	5%	t(24)=4.5972	0.0001
		Male	17%	4%		
	Academic Year Graduating Seniors Survey Summary Reports published between 2009 and 2021	Female	40%	6%	t(24)=4.7822	<0.0001
		Male	29%	6%		

A construct of the national aggregate data helped us compare the results in our medical campus to the results reported by other osteopathic medical schools. During the past decade, the percentage of female and male students expressing an interest in practicing in underserved/shortage areas at the time of matriculation has continually risen every year since 2009 (Figure 3A). Analysis of the national aggregate data between 2009-2022 shows the percentage of female students that had selected "Plans to practice in underserved/shortage area"* *was higher both during matriculation (M=51%, SD=4%) and graduation (M=40%, SD=4%) compared to males (matriculation: M=40%, SD=5%; graduation: M=33%, SD=4%) showing significant differences (matriculation: t(24)=6.7, p<0.0001; graduation: t(24)=5.4, p <0.0001). Those respondents who plan to practice in an underserved/shortage area evolve due to a number of circumstances during the progress of medical education. At the time of graduation (four years later), entering female cohorts matched to graduating female cohorts four years later responded differently (Figure 3A, green). Entering male cohorts matched to graduating male cohorts four years later also responded differently (Figure 3A, yellow). Interest in practicing in underserved/shortage areas most closely aligned for both cohorts between 2020 and 2021 (Figure 3A, green and yellow). Although, the interest was still higher for entering female students at the time of matriculation (solid dots; green) than at the time of graduation (circles; green). For male cohorts, both points (entering and at the time of graduation) converge during this period (2020-2021). It is possible that the larger percentage increase seen during the 2020 and 2021 period for female and male cohorts seen in Figure 3A may have been impacted by the global pandemic (March 2020).

Because more females in our survey strongly agreed or agreed with the statement "I will likely pursue a residency in primary care",* *we compared the findings to the national aggregate data. The percentage of students who plan to practice in primary care specialties is found in satisfaction instruments used for entering and graduating osteopathic medical students, where the data is also separated according to gender [9,10]. A construct of the data helped us compare the data in our campus to that of the national aggregate data with regard to interest in primary care. A higher percentage of females during matriculation (M=25%, SD=5%​​​​) and graduation (M=40%, SD=6%) had selected "Plans to practice in primary care specialties", as compared to males (matriculation: M=17%, SD 4%; graduation M=29%, SD=6%) being statistically different (matriculation: t(24)=4.6, p=0.0001; graduation: t(24)=4.8, p<0.0001)​​. Unlike the data construct in Figure 3A, which suggests interest in practicing in underserved/shortage areas fades from matriculation to the time of graduation for both genders, the interest in primary care increases at the time of graduation (circles) for females (Figure 3B; green) and males (​​Figure 3B; yellow) compared to the time of matriculation (solid dot). It is possible that the largest percentage point increase seen for entering students (solid dots) in 2021, for both female and male cohorts, may have also been impacted by the global pandemic (March 2020).

## Discussion

Participation in the voluntary surveys decreased with the progression of medical education. The highest participation for both genders occurred during the first week of medical education (Table 1) compared to participation during the first week of the third year of medical education (Table 2). This is not surprising as medical students realign their priorities as the availability of time to complete tasks becomes limited throughout their medical education. At the college level prior to starting medical school, student background characteristics (family income, gender, and plans for interacting with a diverse set of individuals) had the greatest impact on intentions to study abroad [11], and undergraduate alumni who studied abroad were more likely to pursue some form of formal education beyond undergraduate studies [12]. Factors such as gender, age, and household income have been associated with interest in global health among nursing and medical students [13]. Medical students interested in pursuing surgery who are female, Black, Hispanic, Indian/Pakistani, multiracial, or had global health experiences were more likely to report intentions to practice in underserved areas [14]. Except for gender, we did not collect other background information. The cohorts we surveyed during the past four years (2019-2022) had been evenly distributed for gender. Like other medical schools which tracked participation in global health electives and had reported more participation from female than male students [15], we also saw that more female students were interested in international medicine clerkship rotation electives before travel abroad came to a halt due to the pandemic.

Our survey results suggest that factors, such as spirituality and/or religion or being competitive for residency match, did not appear to influence responses as those did not rank higher for female respondents (Tables 1 and 2). A greater percentage of our female participants ranked as "very important" to attributes such as developing communication skills to serve foreign-born patients and/or learning about cultural diversity and broadening their worldview. However, helping the underserved ranked the highest (Figure 2). Like the national aggregate data (plans for serving in underserved/shortage areas, Figure 3A), the percentage of females at the time of matriculation and at the time of graduation was higher compared to male cohorts (Figure 3A). The declining interest in practicing in underserved/shortage areas at the time of graduation is unclear but could be related to several factors, such as a competitive residency matching process, or accumulated debt, burnout, or even declining empathy developed during the progress of medical education. For example, studies have reported a progressive decline in empathy does occur throughout medical education [16], and more research will be needed to determine how these and other factors relate to interest in practicing in underserved/shortage areas after graduation. 

Medical students who self-identified from socioeconomically disadvantaged backgrounds were more likely to explore careers in underserved care and show interest in primary care [17]. Female gender has been associated with primary care intentions that were more likely to evolve during clinical training [18]. Graduates of DO schools are also more likely to match into primary care [3], and female students are even more likely to enter into primary care practice [19]. Our construct of the national aggregate data (Figure 3B) shows the percentage of students who matriculated in DO programs with intent to pursue primary care increased later during graduation. A spike in interest in primary care was seen in both genders in 2021, with a higher percentage of females, which could be related to the onset of the COVID-19 pandemic (Figure 3B). The COVID-19 pandemic further raised awareness of the disparities in health and access to care by minority populations, such as the female population of the United States [2]. The pandemic also transformed the international medicine elective landscape by adding more challenges to travel abroad [20]. Analysis of the national aggregate data shows that interest in practicing in the underserved/shortage areas and plans to practice primary care may not be dependent on each other, and there may be other determining factors (Figure 3). Interest in primary care and gender has been associated with higher empathy as well, and although empathy has been shown to decline throughout medical education [16], the reasons for it are still unclear. 

Global health is the collaborative transnational research and action for promoting health for all [21], and opportunities in international medicine provide unique experiences and perspectives in the underserved care for vulnerable populations domestically and worldwide. Completing global health activities in a developing country is likely experienced differently by medical students based on gender [8] because these experiences bring a unique sense of situational awareness that may or may not relate to the previous experiences of the medical student. In the early years of our medical program, we described the challenges in initially supporting global service medical trips due to financial and safety concerns as well as the importance of cultural preparation [8]. However, there are benefits, and those can be viewed as opportunities that facilitate critical self-exploration because these unique experiences promote self-awareness and the ability to recognize and appreciate one's perspective, understandings, values, and personal biases [22]. Unfortunately, travel abroad is often student self-funded and can be costly [23], which can further discourage medical student participation since students are already accumulating substantial debt throughout their medical education and training (Tables 1 and 2). International medicine clerkship elective rotations are not considered core academic programming within medical school curriculums in the United States [24] and, therefore, are not well-funded enough to encourage medical student interest in such activities. Global health activities decreased for graduates from allopathic medical programs [25]. Though during the past decade, there has been an apparent increase in the number of medical schools with structured global health programs, with half of them offering field experiences [26], this still has not adequately addressed the overall desire and intentions of many medical students who indicate an interest in participating in global health activities at the time of matriculation. If a positive correlation exists between global health activities and later interest in primary care, then perhaps nationally consistent global health learning outcomes for medicine could be developed, like those for other health sciences professions [27,28].

These results represent data collected from one osteopathic medical school and may not be representative of all other osteopathic medical schools. Age, ethnicity, and whether participants in our cohorts were first-generation college graduates or first-generation citizens were not collected either. Data from satisfaction instruments completed after 2022 were not made available by AACOM at the time of this study. The results we report here also did not include national data from allopathic medical schools due to differences in the wording of items found in those satisfaction instruments. 

## Conclusions

Medical students, particularly females, who complete global health experiences may be more motivated to pursue primary care. First-year female participants expressed more interest in being prepared to work in global health, functioning in international healthcare settings, developing communication skills to better serve foreign-born patients, and/or gaining clinical experiences internationally. An investment in global health activities and travel abroad during medical education and training could prove worthwhile and serve as a major driver for motivation in primary care, which would potentially serve the needs of American patients, especially those from the healthcare resource-scarce, socioeconomically disadvantaged populations. As efforts for a solution to the primary care physician shortage continue, in this study, we found that most of our first-year female survey participants had a stronger interest in a primary care residency program compared to the male participants.

## Appendices

**Table 5:** Global Health Survey questions ACOM - Alabama College of Osteopathic Medicine

Global Health Survey ACOM							
Q1 With which of the following do you primarily identify?							
Female	Male			Neither		Prefer not to answer	
Q2 Which of the following describes your past experiences? (Please select all that apply).							
1. I have never traveled outside the USA.							
2. I have traveled outside the USA for personal reasons.							
3. I have traveled outside the USA as part of an educational experience.							
4. I have traveled outside the USA as part of mission work related to my place of worship.							
5. I have traveled outside the USA as part of a medical service trip.							
6. I have lived outside the USA.							
7. I have studied another language other than English.							
8. I have achieved fluency in a language other than English.							
Q3 For each of the following items, please rate the relative importance of each item as it relates to your career in medicine.							
		Not at all important	Somewhat important		Important		Very important
Being prepared to work in global health							
Being able to function as physician in international healthcare settings							
Being able to function as physician in underserved areas in other countries							
Learning a language other than English							
Developing communication skills to better serve foreign-born patients							
Gaining clinical experiences in international settings							
Broadening my worldview							
Learning more about cultural diversity							
Helping the underserved							
Learning how medicine is practiced outside the USA							

Q4 For each of the following items, please select the response that best reflects your level of agreement.					
	Strongly disagree	Disagree	Neutral	Agree	Strongly agree
I would like to participate in a global health experience sometime during medical school.					
I would like to engage in international travel while in medical school.					
I would like to travel internationally after I finish medical school.					
I would like to volunteer as a physician in international settings after I complete medical school.					
My religious and/or spiritual community encourages its members to serve on international mission trips.					
Medical students in the USA need to experience how medicine is practiced internationally.					
Medical students in the USA need to learn how to serve patients from other countries.					
International travel provides medical students with career advantages.					
An international experience during medical school will make me more competitive for residency application.					
I will likely pursue a residency in primary care (i.e., family medicine, general internal medicine, or pediatrics).					
Medical students in the USA need to learn how to serve patients from other ethnic cultures.					

Q5 For each of the following items, please rate the extent to which they would prevent you from traveling.			
	This factor would likely discourage me from traveling internationally	This factor would neither discourage nor encourage me to travel internationally	This factor would encourage me to travel internationally
Unanticipated risks/adventure			
Living conditions different from USA			
Foods different from those in USA			
Cultural norms different from those in USA			
Risk of disease			
Risk of injury			
Cost of travel			
Being in situations where I have only myself to rely on			
Q6 Please use this space to provide additional comments regarding your perspectives on global health.			
